# Measuring Burnout Among University Students: Factorial Validity, Invariance, and Latent Profiles of the Italian Version of the Maslach Burnout Inventory Student Survey (MBI-SS)

**DOI:** 10.3389/fpsyg.2018.02105

**Published:** 2018-11-12

**Authors:** Igor Portoghese, Michael P. Leiter, Christina Maslach, Maura Galletta, Fabio Porru, Ernesto D’Aloja, Gabriele Finco, Marcello Campagna

**Affiliations:** ^1^Dipartimento di Scienze Mediche e Sanità Pubblica, Università degli Studi di Cagliari, Cagliari, Italy; ^2^School of Psychology, Deakin University, Melbourne, VIC, Australia; ^3^Department of Psychology, University of California, Berkeley, Berkeley, CA, United States; ^4^Department of Public Health, Erasmus University Medical Center, Rotterdam, Netherlands

**Keywords:** student burnout, Maslach Burnout Inventory-Student Survey, factorial validity, invariance, latent profiles

## Abstract

Burnout has a long tradition of studies in the workplace and recently researchers suggested burnout is also rising among university students. The Maslach Burnout Inventory (MBI) is considered a valid measure of burnout. However, the student version of the MBI (MBI-SS) has received limited empirical support. The aim of this paper is to analyze the factorial validity, invariance, and latent profiles of the Italian version of the MBI-SS in a sample university students. A total of 7757 Italian university students participated in an online cross-sectional survey. Results from explorative and confirmatory factor analyses showed acceptable fits for the Italian version of the MBI-SS. In addition, multigroup analyses supported full-metric invariance of MBI-SS within gender and academic level (bachelor vs. master). Finally, results from latent profile analysis showed that a three latent profile model was the better solution for the data: (a) burned-out (high levels of exhaustion, cynicism (CY), and low professional efficacy (PE); *n* = 2665, 34.2%); (b) overextended (high levels of exhaustion, moderate other, *n* = 3953, 51.0%); and (c) engaged (moderate exhaustion, low CY, and high PE, *n* = 1149, 14.8%). The resulting three-profile solution in the present study partially agrees with a prior study as it replicated three of the five-profile solution identified. In sum, we suggest that the MBI-SS is valid and reliable and represents a robust instrument for the measurement of burnout among Italian speaking university students.

## Introduction

We live in a knowledge-based society where economic growth and social development depend principally on knowledge. In this sense, higher education is one of the most important sectors of our society (Alarcon et al., 2011). Despite education’s importance, university “students today run the risk of not completing their postsecondary education” (Alarcon et al., 2011, p. 211). Specifically, there are increasing global concerns about the mental health of university students (Stallman, 2010) and the high levels of stress and burnout associated with dropout (Deary et al., 2003; Weckwerth and Flynn, 2006; Lin and Huang, 2014; Stallman and Hurst, 2016).

The last decade witnessed an increased global interest in studying and promoting mental health of students. Accordingly, attending university could be(come) a stressful experience for some students (Chambel and Curral, 2005; Salanova et al., 2010; Stallman, 2010; Shin et al., 2011) due to high academic demands; attending classes; respecting deadlines; balancing of study, work, and personal life; and financial pressures (Ryan et al., 2010; Hamaideh, 2011). Many studies have found an increased risk of burnout among students (Schaufeli et al., 2002a; Yang, 2004; Zhang et al., 2007; Dyrbye et al., 2010; Alarcon et al., 2011). In turn, this risk is linked to reduced academic performance (Schaufeli et al., 2002a), low self-efficacy (Yang and Farn, 2005; Edwards et al., 2010; Moneta, 2011), perceived workload (course load; Jacobs and Dodd, 2003; Robins et al., 2015), reduced coping effectiveness (Gan et al., 2007), and suicidal ideation (Dyrbye et al., 2008). Additionally, Lin and Huang (2014) postulated that student burnout may influence students’ relationships with their university and with their fellow students and academic staff.

Despite recent research showing long-term consequences of student burnout, research has rarely considered it as a contributor to work burnout (Dyrbye and Shanafelt, 2011). The proposition that burnout at work has its roots in students’ academic studies increases the urgency of understanding the phenomenon (Dyrbye et al., 2006). The general definition of job burnout considers it as a psychological syndrome of three types of feelings defined as emotional exhaustion (EX), cynicism (CY), and reduced personal accomplishment (Maslach and Jackson, 1981; Maslach et al., 2001). According to Leiter and Maslach (2005), “emotional exhaustion is related to workers’ experience of stress reducing workers’ initiative while progressively limiting their capacity for demanding work” (p. 50). CY refers to detachment from people and work (partially) in reaction to exhaustion and (partially) in reaction to mismatches with the work environment (Maslach et al., 2017). Finally, the third component, perceived professional inefficacy, refers to a state of ineffectiveness and the loss of confidence in own (working) abilities (Maslach et al., 2001).

Based on this theoretical framework, Schaufeli et al. (2002a) proposed that, among students, “burnout refers to feeling exhausted because of study demands, having a cynical and detached attitude toward study, and feeling incompetent as a student” (p. 465).

According to Maslach and Leiter (2016), the MBI “has been considered the standard tool for research” in the burnout field (p. 104). It reflects the original three-dimensions theorization[where (a) emotional “exhaustion is measured by items that refer to fatigue but do not make direct reference to other people as the source of those feelings; (b) cynicism reflects indifference or a distant attitude toward work in general, not necessarily with other people; and (c) professional efficacy (PE) has a broader focus compared to the parallel original MBI scale, encompassing social and nonsocial aspects of occupational accomplishments” (Schaufeli et al., 2002b, p. 465).

In the 4th edition of the manual, Maslach et al. (2017) proposed a version of the MBI-GS developed to measure burnout in college and university students. According to Maslach et al. (2017), the psychometric properties of MBI-GS for Students (MBI-SS) are not yet well documented. Schaufeli et al. (2002a) examined the factorial validity and invariance of the MBI-SS in three different countries: Spain, Portugal, and Netherlands.

The first objective of this study is to investigate the factorial validity, invariance, and reliability of the MBI-SS among Italian university students. Furthermore, we examined factorial invariance of the MBI-SS across gender and university level (bachelor vs. master degree).

In many areas of organizational research, the study of unobserved configurations or patterns of observed individual (also known as latent classes) has a long tradition (Wang and Hanges, 2011). While the use of latent profile analysis (LPA) in social and behavioral sciences has increased in recent years, the application of this technique has been slower in burnout research. In summary, it is as the person-centered approach that explores (latent) differences in the response patterns allowing researchers to identifying and understanding unobserved distinct typologies (profiles) of people (Collins and Lanza, 2010).

According to Morin et al. (2011), these distinct profiles may “represent classification systems designed to help categorize individuals more accurately into qualitatively and quantitatively distinct profiles” (p. 59). Furthermore, LPA is a sophisticated statistical procedure that offers advantages over the classical cluster analyses (K means cluster analysis; Vermunt and Magidson, 2002; Magidson and Vermunt, 2004) allowing researchers to compare alternative models with various fit statistics. In fact, in traditional cluster analysis, researchers follow an arbitrary cluster allocation criterion (Wang and Hanges, 2011).

Recently, Mäkikangas and Kinnunen (2016) suggested that adopting a person-oriented methodology (such as LPA) may expand our knowledge concerning how the three burnout symptoms “combine together at the intraindividual level by forming different burnout types or patterns” (p. 13).

Recently, Leiter and Maslach (2016) used the LPA approach to identify patterns of burnout among workers. They found five patterns (profiles), three of which exist between the endpoints of burnout and engagement. The most straightforward patterns are scoring positively on all three subscales versus scoring negatively on all three subscales. More specifically, Leiter and Maslach (2016) rooted on the conceptual framework of a continuum between burnout and engagement (Leiter and Maslach, 1998) and assumed that work engagement is characterized by energy, involvement, and efficacy which are considered the direct opposites of the three burnout dimensions exhaustion, CY, and lack of PE, respectively. According to Leiter and Maslach (2016), focusing on these three core qualities has the advantage of putting burnout and engagement in the same framework.

Furthermore, given that these three qualities are not perfectly correlated, unique combinations of the three qualities (e.g., positive on two; negative on one) define psychological connections with work that are distinct from the overall positive engagement and the overall negative burnout. From this perspective, an “engaged” profile entails low levels of exhaustion or CY with high PE. On the contrary, a burnout profile entails high exhaustion, high CY, and high PE (Leiter and Maslach, 2016).

The five profiles Leiter and Maslach (2016) identified among workers are: (1) burnout (high exhaustion, high CY, and low PE), (2) disengaged (high CY, moderate exhaustion, and moderate PE), (3) overextended (high exhaustion, moderate CY, and moderate PE), (4) ineffective (low PE, moderate exhaustion, and moderate CY), and (5) engagement (low exhaustion, low CY, and high PE). An ineffective profile is specifically valuable in defining a psychological connection with work that differs from engagement but does not reflect the distress inherent in negative scores on exhaustion and/or CY. These are people living a dull but okay work-life. Differentiating overextended and disengaged allows a more precise differentiating of distressed stated based on the core qualities of exhaustion and CY.

In fact, concerning the academic context, the only study available that adopted a person-oriented approach to capture study burnout was conducted by Salmela-Aro and Read (2017). Unlike Leiter and Maslach (2016); Salmela-Aro and Read (2017) adopted a different perspective assuming that burnout and engagement are distinct. In this sense, they developed an *ad hoc* measure (Study Burnout Inventory) for investigating student burnout and assessed student’s engagement developing an *ad hoc* measure adapted from the Utrecht Work Engagement Scale (UWES-S) originally developed by Schaufeli et al. (2002a). In their study, Salmela-Aro and Read (2017) identified four profiles among students from polytechnics and universities: engaged (positive engagement and low burnout symptom), engaged-exhausted (students showing EX simultaneously with academic engagement), inefficacious (heightened inadequacy), and burned-out (high CY and inadequacy and very low academic engagement).

Thus, the second objective was to use LPA, to classify different patterns of scores on the three MBI-SS subscales. Consistent with Wang and Hanges (2011), we argue that adopting a person-centered approach, this study can provide several important insights into the academic burnout research and, as such, is essentially exploratory.

## Materials and Methods

### Participants and Procedure

The Italian higher education system is organized in three cycles: (1) first cycle (first degree∖bachelor level) – undergraduate studies with a minimum length of 3 years; (2) second cycle (second degree∖master level) – graduate studies with a length of 2 (following in the previous degree)–6 years (for example, medicine and surgery); and (3) third cycle – postgraduate studies (with a minimum length of 1 year).

Participants in this study were undergraduate (bachelor level; *n* = 4723) and graduate (master level; *n* = 3034) Italian students. In total, 7757 university students participated in this study. The convenience sample was recruited through a public announcement at electronic learning platform for students and university students’ associations’ web platforms that contained an invitation of participating in a “Health Promoting University” survey. The online survey was implemented with Limesurvey. Specifically, the survey’s homepage reported information about the study purpose, a general description of the questionnaire, including information about risks and benefits of participation. Also, the time necessary to complete the survey and privacy policy information were reported.

Totally 75.3% was female and 24.7% was male; the mean age was 22.6 years (*SD* = 3.1). 60.9% of students were enrolled in bachelor level courses and 39.1% in master level courses.

### Measures

The Italian version of the MBI-SS (Schaufeli et al., 1996) was back-translated in accordance with Brislin’s procedure (1970). In a second stage, two native English-speaking authors back-translated it. This version was not different from the original one in terms of content and meaning.

The MBI-SS is made up of 15 items that constitute three scales: EX (five items; example item: “*I feel used up at the end of a day at university*”), CY (four items; example item: “*I doubt the significance of my studies*”), and PE (six items; example item: “*During class I feel confident that I am effective in getting things done*”). All the items are scored by using a seven-point Likert scale [from 0 (never) to 6 (always)].

### Statistical Analyses

All statistical analyses were performed with R (R Core Team, 2017) and Rstudio (RStudio Team, 2015). The semTools package (semTools Contributors, 2016) and lavaan (Rosseel, 2012) were used for reliability estimates, confirmatory factor analyses (CFAs) and measurement invariance.

The analyses were conducted in three stages. In the first stage, an explorative factor analysis (EFA) with principal axis factor (PAF) with varimax rotation was performed. We used the Horn’s parallel analysis for factor retention. The internal consistency was assessed via Cronbach’s alpha. To check the factor structure of the Italian version of the MBI-SS, a series of CFAs were performed.

Mardia’s test revealed multivariate non-normality (multivariate kurtosis = 62.18, *p* < 0.0001), thus analyses were performed with robust maximum likelihood (MLM; Brown, 2014).

We compared three alternative models: the one-factor model, in which all 15 items were assessed as one common scale of burnout, a two-factor model where exhaustion and CY were considered as one-factor, and the three-factor model, in which items were divided into three factors reflecting the three subscales of burnout. The CFA results were evaluated by using several indicators: S-Bχ^2^, Robust Satorra–Bentler scaled test χ^2^ (Satorra and Bentler, 1994); the robust root mean square error of approximation (rRMSEA); the standardized root mean square residual (SRMR); the robust comparative fit index (rCFI); and the robust Tucker Lewis index (rTLI). For rCFI and rTLI, a score >0.90 is considered acceptable. For the rRMSEA, 0.05 is considered a good fit and 0.08 a fair fit (Marsh et al., 2004a,b).

In the second stage of analysis, we performed a series of multi-group CFAs for investigating the measurement invariance of the MBI-GS Student. We performed a series of multi-group CFAs. We started with testing configural invariance (Model 0), which represents the least constrained model. Then, we tested for metric invariance (Model 1) by constraining factor loadings. In testing scalar invariance (Model 2), we constrained factor loadings and item intercepts. In testing uniqueness invariance (Model 3), we constrained factor loadings, item intercepts, and residual item variances/covariances. Finally, in testing structural invariance (Model 4), we constrained factor loadings, item intercepts, and factor variances/covariances. Each model is nested within its previous model that were compared by using the chi-square (χ^2^; Bentler, 1990). However, the χ^2^ is sensitive to sample size, especially large samples (Cheung and Rensvold, 2002; French and Finch, 2006; Chen, 2007; Meade et al., 2008). In this sense, additional criterion should be considered in comparing nested models.

Specifically, in the present study, we used the change in CFI, RMSEA, and SRMR indices. The criteria we followed for testing invariance considered were: ΔCFI ≤ -0.02 (Meade et al., 2008; Rutkowski and Svetina, 2014), ΔRMSEA ≤ 0.015, and ΔSRMR ≤ 0.03 for tests of factor loading invariance (Chen, 2007; Meade et al., 2008) and ΔCFI ≤-0.01, RMSEA ≤ 0.015, and SRMR ≤ 0.01 for test of scalar invariance (Chen, 2007).

The third stage of analysis involved conducting LPA. Starting from the best measurement invariance model from the multi-group CFA, we specified models from two to six latent profiles. In deciding the optimal number of profiles (McLachlan and Peel, 2000), we considered (a) the Bayesian Information Criterion (BIC; Schwarz, 1978) where lower values for this statistic represent a superior fit; (b) the Bootstrap Likelihood Ratio Test (BLRT; McLachlan and Peel, 2000), where a significant *p*-value will indicate that a latent profile solution is better than a solution with less profiles (McLachlan and Peel, 2000). We evaluated classification accuracy by considering the Entropy (Nylund et al., 2007), with higher values (from 0 to 1) representing smaller classification errors.

Finally, we used an arbitrary statistical cut-off criterion based on percentile 33rd and percentile 66th to identify, respectively, low, moderate, and high levels.

## Results

### Exploratory Factor Analysis

The dataset was plotted into random training and test samples. We used the training sample (*n* = 3879) for the exploratory analysis. We first examined model assumptions. Results from parallel analysis (5000 parallel data sets using 95th percentile random eigenvalue) found eight latent factors for retention. The eigenvalues for the first three factors generated by the PAF exceeded those generated by the random datasets. The eight-latent factor solution was considered problematic and not theoretically founded. Specifically, the eigenvalues of several factors were considered to be trivial. This is in line with Timmerman and Lorenzo-Seva (2011) study that showed how Horn’s Parallel Analysis power to detect minor factors increases with increasing sample size (overfactoring). Thus, according to the original version of the MBI-SS, a three-factor solution was analyzed. Thus, we conducted a PAF analysis with varimax rotation on the 15 items. The three-factor solution explained 46.3% of the variance in the three facets. After rotation, the factors were interpreted as EX, CY, and PE (Table 1).

**Table 1:** Factor pattern matrix for the Italian version of the MBI-SS.

	Emotional exhaustion		Cynicism		Professional efficacy	
	EFA	CFA	EFA	CFA	EFA	CFA
Exh5	0.75	0.85^∗^	–	–	–	–
Exh3	0.77	0.66^∗^	–	–	–	–
Exh1	0.74	0.79^∗^	–	–	–	–
Exh2	0.73	0.60^∗^	–	–	–	–
Exh4	0.67	0.68^∗^	–	–	–	–
Cyn7	–	–	0.72	0.90^∗^	–	–
Cyn6	–	–	0.69	0.87^∗^	–	–
Cyn8	–	–	0.67	0.58^∗^	–	–
Cyn9	–	–	0.57	0.49^∗^	–	–
PE14	–	–	–	–	0.67	0.59^∗^
PE13	–	–	–	–	0.64	0.67^∗^
PE15	–	–	–	–	0.63	0.56^∗^
PE11	–	–	–	–	0.62	0.62^∗^
PE12	–	–	–	–	0.45	0.56^∗^
PE10	–	–	–	–	0.41	0.55^∗^


*EFA, Explorative Factor Analysis; *n* = 3879. CFA, Confirmative Factor Analysis; *n* = 3878. Loadings below —0.35— have been suppressed. ^∗^*p* < 0.01.*

### Confirmatory Factor Analysis

Based on the results from the EFA, we tested four models on the test sample (*n* = 3879; Table 2): a one-factor model, a two-factor model where exhaustion and CY were considered as one-factor, a three factor model, and a three-factor model with adjustments made according to error theory.

**Table 2:** Fit indices of the MBI-SS from the CFA.

Model	S-Bχ^2^	*df*	ΔS-Bχ^2^	Δ*df*	*p*	rCFI	rTLI	rRMSEA	SRMR
One-factor model	9005.89	90				0.55	0.47	0.174	0.121
Two-factor model (exhaustion and CY as one-factor)	5081.20	86	3924.69	4	<0.001	0.75	0.70	0.133	0.098
Three-factor model	2808.93	87	6196.96	3	<0.001	0.86	0.84	0.098	0.063
Three-factor model nested (MIs inspection)	1719.17	84	1089.76	3	<0.001	0.92	0.90	0.076	0.055


**n* = 3878; S-Bχ^*2*^ = Satorra–Bentler scaled chi-square, rCFI, robust comparative fit index; rTLI, robust Tucker–Lewis index; RMSEA, robust root mean square error of approximation; SRMR, standardized root mean residual.*

Fit indices for the unidimensional model S-Bχ^2^(90) = 9005.89, rCFI = 0.55, rTLI = 0.47, RMSEA = 0.174, and SRMR = 0.121 did not provide a good fit to the data. Then we considered the three-factor model as theorized by Maslach et al. (2017). Fit indices suggested a poor fit to the data S-Bχ^2^(87) = 2805.93, rCFI = 0.86, rTLI = 0.84, rRMSEA = 0.098, and SRMR = 0.063. Then, inspecting the modification indices (MIs) we found three residual correlations within the three sub-scales as potential sources of model misfit. We freed the covariance between errors of items Exh2 (“*I feel used up at the end of a day at university*”) and Exh3 (“*I feel tired when I get up in the morning and I have to face another day at the university*”), residual correlation *r* = 0.39; Cyn3 (“*I doubt the significance of my studies*”) and Cyn4 (“*I have become more cynical about the potential usefulness of my studies*”), residual correlation *r* = 0.39; PE5 (“*During class I feel confident that I am effective in getting things done*”) and PE6 (“*I believe that I make an effective contribution to the classes that I attend*”), residual correlation *r* = 0.29. These measurement residual covariances represent random measurement error in item responses. In this sense, residual covariances are not because of conceptual reasons – misspecification of the model – but because of technical aspects, mainly because of their high degree of content overlap (Cole et al., 2007). Thus, we included these residual correlations in the model. The revised model fit the data well, S-Bχ^2^(84) = 1719.17, rCFI = 0.92, rTLI = 0.90, rRMSEA = 0.076, SRMR = 0.055. The χ^2^ difference test was significant, ΔS-Bχ^2^(3) = 1089.76, *p* < 0.001.

All standardized factor loadings differed significantly from zero (*p* < 0.001). All three subscales had good internal consistency: EX = 0.86, CY = 0.82, and PE = 0.77. Correlations between the three latent burnout factors were as follows: 0.52 between EX and CY, -0.36 between EX and PE, and -0.52 between CY and PE. Thus, the Italian version of the MBI-SS can be considered a three-component measure with three separate subscales.

### Measurement Invariance

#### Courses Invariance Assessment

Next, a series of multi-group CFAs across university courses groups (bachelor vs. master) and gender were conducted to provide evidence of the MBI-SS measurement invariance across different groups. The results of the model fit tests and the model comparison are summarized in Table 3.

**Table 3:** Test of invariance of the proposed three-factor structure of the MBI-SS between bachelor (*n* = 4723) and master (*n* = 3034) students, and female (*n* = 5843) vs. male students (*n* = 1914): results of multigroup CFAs.

Model	S-Bχ^2^	*df*	CFI	RMSEA	SRMR	Nested model	ΔCFI	ΔRMSEA	ΔSRMR
**Bachelor vs. master students**					
Bachelor students	2652.57	84	0.909	0.080	0.058				
Master students	1400.92	84	0.931	0.072	0.052				
M0. Configural invariance	4053.54	168	0.918	0.077	0.056				
M1. Metric invariance	4097.92	180	0.917	0.075	0.058	M1–M0	-0.001	-0.002	0.002
M2. Scalar invariance	4644.33	192	0.906	0.077	0.056	M2–M1	-0.011	0.002	-0.002
M2. Partial scalar invariance	4335.11	189	0.912	0.075	0.058	M2b–M1	0.006	-0.002	0.002
M3. Uniqueness invariance	4772.15	207	0.903	0.075	0.062	M3–M2b	-0.009	0.000	0.004
M4. Structural invariance	5004.68	209	0.899	0.077	0.066	M4–M3	-0.004	0.002	0.004
**Female vs. male students**					
Female students	2845.92	84	0.923	0.075	0.055				
Male students	1113.08	84	0.912	0.080	0.056				
M0. Configural invariance	3959.00	168	0.920	0.076	0.055				
M1. Metric invariance	4036.74	180	0.919	0.074	0.057	M1–M0	-0.001	-0.002	0.002
M2. Scalar invariance	4294.69	192	0.914	0.074	0.059	M2–M1	-0.005	0.000	0.002
M3. Uniqueness invariance	4389.34	207	0.912	0.072	0.059	M3–M2	-0.002	-0.002	0.000
M4. Structural invariance	4471.07	210	0.910	0.072	0.061	M4–M3	-0.002	0.000	0.002


**df*, degrees of freedom; CFI, comparative fit index; TLI, Tucker–Lewis Index; RMSEA, root mean square error of approximation; SRMR, standardized root mean square residual.*

First, a multi-group CFA was conducted on the bachelor and master groups. As shown in Table 3, the unconstrained model considered for testing configural invariance (M0) fit the data well across bachelor (*n* = 4723) and master (*n* = 3034) groups: S-Bχ^2^(168) = 4053.80, CFI = 0.918, RMSEA = 0.077, and SRMR = 0.056. All loadings were significantly different from zero (*p* < 0.01). Next, results from metric invariance showed that the model (M1) also fit the data well: ΔCFI = -0.001, ΔRMSEA = -0.002, and ΔSRMR = -0.002. Thus, the additional constraints we imposed on this model did not result in significant change in its fit.

Next, we tested for scalar invariance (M2) constraining item intercepts to be equal across groups. Results showed a significant drop in model fit (ΔCFI = -0.011) rejecting full scalar invariance. Thus, we tested for partial invariance. By inspecting MIs, we found that one item from the CY subscale (“*I doubt the significance of my studies*”) and two items from the PE subscale (“*I have learned many interesting things during the course of my studies*” and “*During class I feel confident that I am effective in getting things done*”) lacked invariance. Then the intercepts of these items were freely estimated (Byrne et al., 1989), partial scalar invariance was supported because the fit of M2b was not significantly different from the fit of M1 (ΔCF = 0.006, ΔRMSEA = -0.002, and ΔSRMR = 0.002).

Next, we tested for uniqueness invariance (M3) constraining factor loadings, item intercepts, and residual item variances/covariances. Results showed that the model also fit the data well: ΔCFI = -0.009, ΔRMSEA = 0.000, and ΔSRMR = 0.004.

Finally, structural invariance (M4) was also supported because adding additional constraints to M3 did not change the fit significantly: ΔCFI = -0.004, ΔRMSEA = 0.002, and ΔSRMR = 0.004.

Next, we performed MGCFAs to test the invariance of the MBI-SS between female and male students. As shown in Table 3, the unconstrained model used to test for configural invariance (M0) fit the data well across female (*n* = 5843) and male (*n* = 1914) groups: S-Bχ^2^(168) = 3959.00, CFI = 0.920, RMSEA = 0.076, SRMR = 0.055. All loadings were significantly different from zero (*p* < 0.01). Next, results from metric invariance showed that the model (M1) also fit the data well: ΔCFI = -0.001, ΔRMSEA = -0.002, and ΔSRMR = -0.002.

Next, we tested for scalar invariance (M2). Results showed that imposing additional constraints did not alter its fit significantly finding support for scalar invariance: ΔCFI = -0.005, ΔRMSEA = 0.000, and ΔSRMR = 0.002. The next step was to test for uniqueness invariance (M3). Results showed that the model also fit the data well: ΔCFI = -0.002, ΔRMSEA = -0.002, and ΔSRMR = 0.000. Thus, imposing additional constraints on this model did not result in significant changes in its fit. Finally, structural invariance (M4) was also supported: ΔCFI = -0.002, ΔRMSEA = 0.000, and ΔSRMR = 0.002.

### Latent Profile Analysis

An LPA on the three dimensions of MBI-SS was performed. Within-profile means and variances of the observed variables were estimated. According to the assumption of local independence in the classical LPA (Marsh et al., 2009), we fixed to zero the residual covariances between the indicators.

We tested for LPA with two–six profiles. As shown in Table 4, a diagonal model with varying volume and shape model (VVI) with three-profile solution was considered as the optimal solution.

**Table 4:** Latent profile models and fit indices.

Model	Log-likelihood	BIC	Entropy	BLRT *p*-value
Two profiles	35,946.453	72,063.076	0.87	0.25
Three profiles	35,578.002	71,335.131	0.87	<0.001


*BIC, Bayesian information criterion; BLRT, bootstrap likelihood ratio test.*

Descriptive statistics of the Italian version of the MBI-SS by profile are presented in Table 5. Profile 1 (*n* = 2665, 34.2%) was labeled as “burned-out” as it had high levels of exhaustion, CY, and low PE. Profile 2 (*n* = 3953, 51.0%) was labeled as “overextended” as it had (moderately) high levels of exhaustion, moderate other. Finally, profile 3 (*n* = 1149, 14.8%) was labeled as “engaged” as it had moderate exhaustion, low CY, and high PE.

**Table 5:** Descriptive statistics MBI scale by profile.

Profile	*n*	Exhaustion	Cynicism	Professional efficacy
		*M* (*SD*)	*M* (*SD*)	*M* (*SD*)
Burnout	2655	4.24 (1.12)	3.42 (1.17)	2.98 (0.97)
Overextended	3953	3.01 (1.19)	1.13 (0.59)	3.84 (0.85)
Engagement	1149	2.56 (1.27)	0.06 (0.11)	4.50 (0.75)
Overall mean (*SD*)	7757	3.36 (1.34)	1.75 (1.49)	3.64 (1.02)


## Discussion

The purpose of this study was to analyze the factorial validity, invariance, and latent profiles of the Italian version of the MBI-SS in a sample of Italian university students. Overall, this examination of responses from a large sample of Italian university students provides support for the structure of the MBI-SS as providing measures of exhaustion, CY, and PE. The CFA concurred with the assignment of items to the three designated subscales with minimal indication of cross-loading of items across the factors.

The MBI-SS emerges as a reliable instrument for measuring academic burnout. The internal consistency of the subscales is good. This study is the first to provide evidence for the psychometric properties of the Italian version of the MBI-SS (Maslach et al., 2017).

As expected, we established metric invariance across genders and academic level (bachelor vs. master). In fact, multigroup CFAs confirmed that the MBI-SS is composed of three distinct components and this structure is (mostly) invariant across the different groups. Our results supported parameter equivalence across gender, but the MBI-SS was significantly different in bachelor versus master level. Specifically, it was not found to be scalar invariant, suggesting that the item CYN3 (“*I doubt the significance of my studies*”) and two items from the PE subscale (“*I have learned many interesting things during the course of my studies*” and “*During class I feel confident that I am effective in getting things done*”) vary by academic level. It is possible that these differences on the aforementioned items reflect differences in expectations and experiences among students. While removing the three problematic items may be considered a possible solution to the lack of scalar invariance, this may remove significant characteristics of the studied constructs. However, as the MBI-SS showed metric invariance, the lack of scalar invariance represents a marginal issue.

Additionally, the profile analysis provided results consistent with those of Leiter and Maslach (2016) in identifying an overextended profile as well as the burnout and engaged profiles. This result strengthens the argument for the MBI as a means of identifying a range of psychological connections with work, not simply as a one-dimensional instrument reflecting solely the extent to which respondent’s experience burnout. The profile analysis identified profiles on the opposite poles of the combined three subscales: burnout (high exhaustion, high CY, low PE) and engagement (low exhaustion, low CY, and high PE). These profiles follow the use of the MBI-SS as a measure of burnout with responses on the negative end reflecting burnout and on the positive end reflecting engagement. The additional overextended profile was partially consistent with a profile identified in Leiter and Maslach (2016) in having high scores on exhaustion combined with moderate scores on the other two subscales. This profile conveys that a recognizable subgroup of students feels exhausted while maintaining moderate levels of involvement (moderate CY) and efficacy in their academic work. The analysis did not identify a disengaged or ineffective profile with negative scores solely on CY and efficacy, respectively. It may be that university students have less susceptibility to this configuration of experiences that appears to be prevalent in working populations. One reason students have high scores on exhaustion due to student life styles that often are less orderly than those of working people. In fact, university life is more compatible with late nights (mainly change in circadian rhythm that delays sleep and wake onset; Lund et al., 2010) than much of working life. Furthermore, concerning the disengaged profile, one source of CY and therefore disengagement is the transition from the idealistic world of university to the real world of many occupations. University life may be more supportive of a person’s sense of efficacy with its relatively short timescale and frequent opportunities for feedback. Students who receive consistently negative feedback are likely to leave university. With both of these factors in play, students are unlikely to experience CY on its only, but only in combination with exhaustion when overwhelmed by demands which would result in burnout rather than disengagement or ineffective.

The other departure from Leiter and Maslach’s (2016) analysis of healthcare employees is that the student sample had over twice as many participants in the burnout profile than in the engaged profile. Among healthcare employees, the engaged profile was four times more populous than the burnout profile. This difference is consistent with the students’ mean on exhaustion (*M* = 3.36, *SD* = 1.34) that is nearly full standard deviation more negative than the exhaustion score for the health care providers (*M* = 2.19, *SD* = 1.49) in Leiter and Maslach (2016). The students’ high score on exhaustion is consistent with the overextended profile being over half of the student sample (*N* = 3953/7757).

### Limitations and Future Directions

There are several limitations to the current research. First, in the current study, we assessed the internal psychometric features of the Italian version of the MBI-SS without referring to content and discriminant validity and test–retest reliability. Future research should address these limitations, investigating the consistency over time of the MBI-SS and its relationship with other variables in a similar fashion as has been done with the workers’ version of the MBI. Second, using MIs with the aim of improving the overall model fit should be taken with carefulness (Cole et al., 2007). In fact, correlating error terms means that covariation may be linked to other not specified issues within the model. However, our decision was substantively meaningful and theoretically reasonable.

Third, as our data are based on a single measurement wave, we were not able to test for test–retest reliability. Future research should address this issue.

## Conclusion

In the present study, we found that the Italian translation of the MBI-SS confirms its three-factor structure and that this structure is invariant for both males and females students and types of academic level (bachelor and master). According to a recent meta-analysis on interventions to reduce stress in university students (Regehr et al., 2013), a limited number of students experiencing academic stress receive treatment and universities should employ preventive interventions. The assessment of psychological stress and burnout should consider the use of internationally validated measures. Thus, the Italian version of the MBI-SS can be administered for measuring academic burnout on Italian-speaking university students.

Finally, findings from the LPA highlight that the overextended profile indicates a sole problem with exhaustion and therefore with managing academic demands. This differs from burnout that reflects a more broad life crisis in addition to the exhaustion/workload issue. Overextended is a simpler problem to address in that it calls for strategies for managing workload. Then, universities need not expend extra resources that would be necessary to help students who are fully burned out.

## Ethics Statement

This study has been conducted in accordance with the recommendations of the local ethic committee at the University of Cagliari. No ethical approval is required in Italy for observational nature studies as they are not defined as medical∖clinical research, referring to the Italian law 211/2003. In fact, the study included non-clinical surveys using non-invasive measures (self-ratings). Furthermore, this study complies with the Declaration of Helsinki in 1995 (as revised in Edinburgh 2000) and with Italian privacy law (Decree No. 196/2003). No treatments or false feedbacks were given, and no potential harmful evaluation methods were used. Participation was completely voluntary, and participants could drop out at any time without any negative consequences. All data were stored only using an anonymous ID for each participant. Written online informed consent to participate in the survey was obtained by clicking on “I accept.”

## Author Contributions

IP, MG, ML, and CM led the literature review and paper drafting work for this paper, and interpreted the findings. IP and MG made contributions in data analysis. FP made contributions in data collection. ED, GF, and MC provided critical revision of the manuscript.

## Conflict of Interest Statement

The authors declare that the research was conducted in the absence of any commercial or financial relationships that could be construed as a potential conflict of interest.
